# Identification of a Key Enzyme for the Hydrolysis of β-(1→3)-Xylosyl Linkage in Red Alga Dulse Xylooligosaccharide from *Bifidobacterium Adolescentis*

**DOI:** 10.3390/md18030174

**Published:** 2020-03-20

**Authors:** Manami Kobayashi, Yuya Kumagai, Yohei Yamamoto, Hajime Yasui, Hideki Kishimura

**Affiliations:** 1 Chair of Marine Chemical Resource Development, Graduate School of Fisheries Sciences, Hokkaido University, Hakodate 041-8611, Hokkaido, Japanbknb626@gmail.com (Y.Y.); 2 Laboratory of Marine Chemical Resource Development, Faculty of Fisheries Sciences, Hokkaido University, Hakodate 041-8611, Hokkaido, Japan; yuyakumagai@fish.hokudai.ac.jp; 3 Laboratory of Humans and the Ocean, Faculty of Fisheries Sciences, Hokkaido University, Hakodate 041-8611, Hokkaido, Japan; hagime@fish.hokudai.ac.jp

**Keywords:** red alga, dulse, xylooligosaccharide, β-(1→3)/β-(1→4)-xylan, Bifidobacterium, GH43, β-xylosidase

## Abstract

Red alga dulse possesses a unique xylan, which is composed of a linear β-(1→3)/β-(1→4)-xylosyl linkage. We previously prepared characteristic xylooligosaccharide (DX3, (β-(1→3)-xylosyl-xylobiose)) from dulse. In this study, we evaluated the prebiotic effect of DX3 on enteric bacterium. Although DX3 was utilized by *Bacteroides* sp. and *Bifidobacterium adolescentis*, *Bacteroides* Ksp. grew slowly as compared with β-(1→4)-xylotriose (X3) but *B*. *adolescentis* grew similar to X3. Therefore, we aimed to find the key DX3 hydrolysis enzymes in *B*. *adolescentis*. From bioinformatics analysis, two enzymes from the glycoside hydrolase family 43 (BAD0423: subfamily 12 and BAD0428: subfamily 11) were selected and expressed in *Escherichia coli*. BAD0423 hydrolyzed β-(1→3)-xylosyl linkage in DX3 with the specific activity of 2988 mU/mg producing xylose (X1) and xylobiose (X2), and showed low activity on X2 and X3. BAD0428 showed high activity on X2 and X3 producing X1, and the activity of BAD0428 on DX3 was 1298 mU/mg producing X1. Cooperative hydrolysis of DX3 was found in the combination of BAD0423 and BAD0428 producing X1 as the main product. From enzymatic character, hydrolysis of X3 was completed by one enzyme BAD0428, whereas hydrolysis of DX3 needed more than two enzymes.

## 1. Introduction

Terrestrial xylan is a β-(1→4)-xylosyl polymer possessing arabinofuranose or glucuronic acid side chains at C2 or C3 positions [1]. These oligosaccharides (XOS) are nondigestible ingredients and have the advantage of stability at a low pH and heat resistance for food industry usage as compared with other types of oligosaccharides [2,3]. XOS are utilized in human gut microbiota and show various beneficial effects on human health [4,5]. XOS as a dietary supplementation improv the blood sugar and lipids in type-2 diabetes mellitus [6]. XOS from birchwood xylan show a favorable effect on human intestinal flora [7]. Namely, in vitro analysis has shown that Bifidobacteria utilizes XOS, whereas *Escherichia coli* and *Clostridium* spp. does not utilize XOS. In addition, in vivo analysis has shown that the growth of *Bifidobacteria* was promoted by 5  g/day. Therefore, XOS have been used as a functional food material, for example, as dietary sweeteners for low-calorie foods [8,9]. 

Marine algae contain polysaccharides with no lignin and less cellulose [10,11]. Most red algae possess sulfated galactan such as agar and carrageenan [12,13], and those oligosaccharides show a prebiotic effect [14]. Among red algae, dulse (*Palmaria palmata*) is known to contain a mix-linked β-(1→3)/β-(1→4)-xylan in the cell wall [15,16,17,18]. These studies have focused on the elucidation of their structure, whereas our previous study showed the method of dulse XOS production [19]. The xylotriose (DX3) possesses β-(1→3)-xylosyl linkage at the nonreducing end. 

Terrestrial β-(1→4)-XOS has been hydrolyzed by β-xylosidase (EC 3.2.1.37), which showed the bifunctional activities of α-l-arabinofuranosidase due to their spatial similarities between D-xylopyranose and L-arabinofuranose. On the one hand, the enzyme character has been well characterized in gut microbiota such as Bifidobacterium and Bacteroides [20]. Most enzymes in these bacteria were intracellular enzymes [21]. The oligosaccharide uptake transporter has been characterized in Bifidobacterium [22]. On the other hand, the enzyme which hydrolyzed β-(1→3)-xylosyl linkage was not reported from gut microbiota. β-(1→3)-Xylosidase was reported from *Streptomyces* sp. SWU10 and *Vibrio* sp. XY-214 [23,24]. These enzymes were classified into the glycoside hydrolase family (GH) 43 and subfamily 11 (*Vibrio* sp. XY-214) and 12 (*Streptomyces* sp. SWU10) [25]. The characterized enzyme activity of GH43_11 and GH43_12 was β-(1→4)-xylosidase and α-l-arabinofuranosidase, respectively, indicating that a key enzyme for hydrolysis of β-(1→3)-xylosyl linkage in gut microbiota was not clear. 

A previous study has shown that the dulse in Japan is abundant in proteins (approximately 40  g/100  g dried dulse) and the major component is phycoerythrin (PE) [26]. The peptides produced from thermolysin hydrolysis showed the inhibitory activity of the angiotensin-I-converting enzyme (ACE) which was found in soluble proteins from many red algae [27,28,29,30,31]. The chromophores from PE showed antioxidant activity [32]. To obtain more functionality of dulse, we prepared xylooligosaccharide from dulse, which possessed the unique structure of β-(1→3)/β-(1→4)-xylotriose (DX3) [19]. In this study, we investigated the prebiotic effect of DX3 on enteric bacteria, showing that *Bifidobacterium adolescentis* grew quickly among the tested bacteria. We then attempted to find the key enzyme for DX3 hydrolysis from *B*. *adolescentis* and to discuss the metabolic pathway of DX3. 

## 2. Results

### 2.1. In Vitro Utilization of XOS by Entric Bacteria

**Figure 1:**
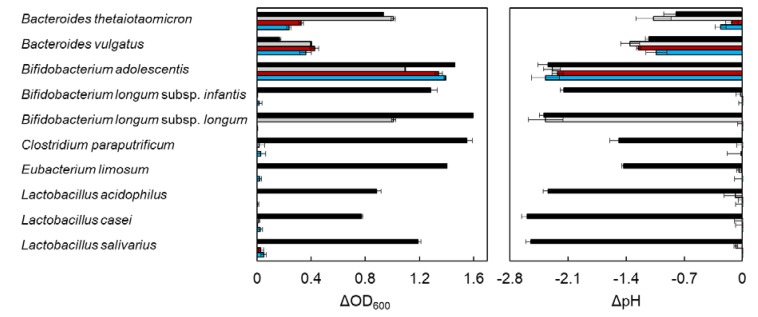
Effect of carbohydrate on bacterial growth and pH. The bacterial strains were cultured at 37 °C for 96 h in PYF (Peptone−Yeast extract−Fildes) medium containing 0.5% carbohydrate samples under anaerobic conditions. The data were obtained as *Δ*OD_600_ and *Δ*pH by the subtraction of each bacterial growth without carbohydrate samples. Error bars indicate SD (n = 3). Growth data of *C*. *paraputrificum* and *E*. *limosum* for β-(1→4)-xylotriose (X3) were not determined. Symbols: black bar, glucose; gray bar, xylose; red bar, X3; and blue bar, β-(1→3)/β-(1→4)-xylotriose (DX3).

DX3 is a xylotriose from red alga dulse, which possesses β-(1→3)-xylosyl linkage at the nonreducing end. This structure differs in β-(1→4)-xylotriose (X3) from terrestrial plant, which is composed of only β-(1→4)-xylosyl linkage. To evaluate the effect of DX3 on entric bacterial growth, ΔOD_600_ and ΔpH were measured after incubation for 96 h using 10 bacteria (Figure 1 and Table S1). The data were obtained by subtraction of the sample without carbohydrates. Glucose (G1) and xylose (X1) were used as positive controls. In the case of Bacteroides thetaiotaomicron, the growth rate of X3 and DX3 was slow as compared with the medium in G1 and X1. X3 was a more suitable carbon source for the growth of Bacteroides sp. than DX3. Bifidobacterium sp. was classified into three types. The growth of B. longum subsp. infantis increased in only G1, and the growth of B. longum subsp. longum increased in G1 and X1 but not in X3 and DX3. The growth of B. adolescentis increased in X3 and DX3 similar to G1. The rest of the species, which did not metabolize xylose, could also not utilize X3 and DX3. The growth of all bacteria tested in this study was accompanied by a decrease in pH, due to the production of short chain fatty acid resulting from the bacterial fermentation [33,34]. Among the tested bacteria, the utilization of XOS between Bacteroides sp. and B. adolescentis differed. Then, we compared the time course growth rate between them. 

### 2.2. Time Course Growth in B. vulgatus and B. adolescentis

**Figure 2:**
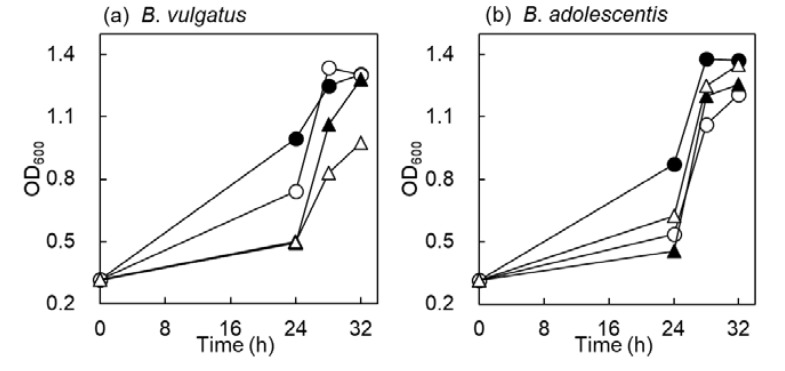
Time course growth rate of *B*. *vulgatus* (**a**) and *B*. *adolescentis* (**b**). Bacteria grow in PYF medium containing 0.5% carbohydrates at 37 °C in anaerobic condition. Symbols: ●, glucose (G1); ◯, xylose (X1); ▲, X3; △, DX3. Mean  ±  standard deviation of three replicate determinations and the error bars were within the symbols.

Growth rate of bacteria in PYF medium containing carbohydrates (G1, X1, X3 and DX3) was monitored at 24, 28, and 32 h (Figure 2). *B. vulgatus* grew quickly in G1 and X1. On the one hand, growth of *B. vulgatus* among XOS was the same up to 24 h, but growth in X3 was fast at 24 to 32 h. On the other hand, *B*. *adolescentis* grew rapidly in G1, and the growth rate was almost the same in X1, X3 and DX3. This result suggested that *B*. *adolescentis* utilized DX3 similar to X3, and the utilization of DX3 in *B*. *vulgatus* was slow as compared with X3. The difference of DX3 and X3 was the linkage at the nonreducing end. Exo hydrolase enzymes generally act on the nonreducing end of carbohydrates [35,36]. This means that *B*. *adolescentis* would possess the key enzyme for DX3 hydrolysis. Therefore, we attempted to determine the enzymes by comparing the related genes from CAZy database. 

### 2.3. Comparison of β-Xylosidase Genes in the Tested Bacterium

**Figure 3:**
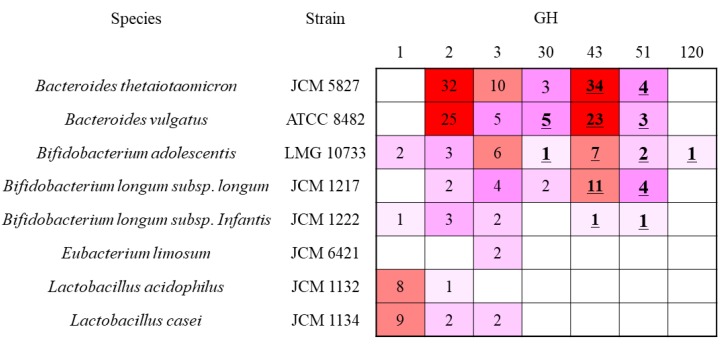
Relationship between bacteria and the number of enzymes containing β-xylosidase (EC 3.2.1.37) in glycoside hydrolase families (GHs). The colors are related to the number of enzymes from pink (low) to red (high). The bold and underlined number means that GHs contain β-xylosidase.

GH was classified into their primary sequences. Therefore, each family possessed enzymes having different substrate specificity. β-Xylosidase (EC 3.2.1.37) was classified into 13 families, and genes of the tested bacteria belonged to GH 1, 2, 3, 30, 43, 51, and 120 (Figure 3). We attempted to find the specific DX3 hydrolysis enzymes from *B*. *adolescentis. B*. *adolescentis* possessed putative 22 β-xylosidase genes. Among them, Blast analysis revealed that two GH1 and six GH3 enzymes were β-glucosidase, and three GH2 enzymes were β-galactosidase. The GH30 enzyme was subdivided into nine subfamilies, and only the subfamily 2 consisted of β-xylosidase. The *B*. *adolescentis* GH30 enzyme was subfamily 2, but two *B. longum* subsp. *longum* GH30 genes were subfamily 5. Within two *Bacteroides* sp., *B*. *vulgatus* possessed two subfamily 2 enzymes. One GH30 from the *B*. *vulgatus* enzyme showed 60% identity with the *B*. *adolescentis* GH30 enzyme. In addition to the low identity between them, *B*. *thetaiotaomicron* did not possess the enzyme, indicating that this is not a key enzyme for DX3 hydrolysis. The activity of GH51 enzyme in the tested bacterium was reported as α-l-arabinofuranosidase. Bacteria, which can or cannot utilize X3 and DX3, also possessed GH51, indicating that this family was not the key enzyme for DX3 hydrolysis. The GH120 enzyme is classified into β-xylosidase, and only found in *B*. *adolescentis* in the tested bacterium. Previous studies have reported that the enzyme preferred longer XOS larger than X4 [33,37,38]. The target XOS in this study is X3. Therefore, we excluded this enzyme, and a more detailed explanation is included in the Discussion section. GH43 is a large family containing 37 subfamilies, and many studies have been performed with the understanding of the relationship between *Bifidobacterium* sp. and XOS [39,40,41,42,43,44,45]. To find the key enzymes for DX3 hydrolysis, the enzymes in GH43 were evaluated. 

### 2.4. β-Xylosidase in GH43 Subfamily

**Figure 4:**
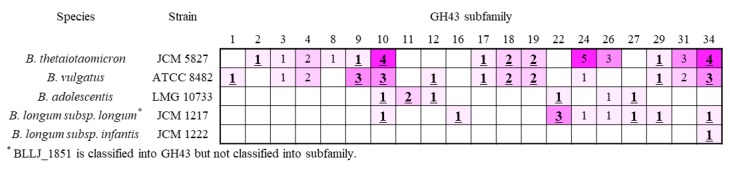
Relationship between bacteria and the number of enzymes in the GH43 subfamily. The colors are related to the number of enzymes from pink (low) to purple (high). The bold and underlined number shows subfamilies contain β-xylosidase.

Among 37 subfamilies in GH43, 21 subfamilies were characterized as β-xylosidase or α-l-arabinofuranosidase (Figure 4). Six of seven *B*. *adolescentis* enzymes showed the enzyme activity, except for subfamily 26. A comparison of the GH43 subfamilies of *B*. *adolescentis* (DX3 metabolic bacterium) to *B*. *longum* sp. (DX3 non-metabolic bacteria), showed that subfamilies 11 and 12 were only found in *B*. *adolescentis*. Although *B*. *vulgatus* also possessed GH43 subfamily 12 (GH43_12), the bacterium also utilized X3 and DX3. From these characteristics, GH43_11 and _12 were the candidates for DX3 hydrolysis. GH43_11 was characterized as β-xylosidase (BAD0428 and BAD1203) and GH43_12 as α-l-arabinofuranosidase (BAD0423). In addition, information about the activity of β-(1→3)-xylosidase from *Streptomyces* sp. (GH43_12) and *Vibrio* sp. (GH43_11) would be a clue for finding β-(1→3)-xylosidase from gut microbiota. GH43_22 (BAD1527) β-xylosidase was also employed for the enzymatic analysis. 

### 2.5. Enzymatic Character of GH43 Enzymes

**Figure 5:**
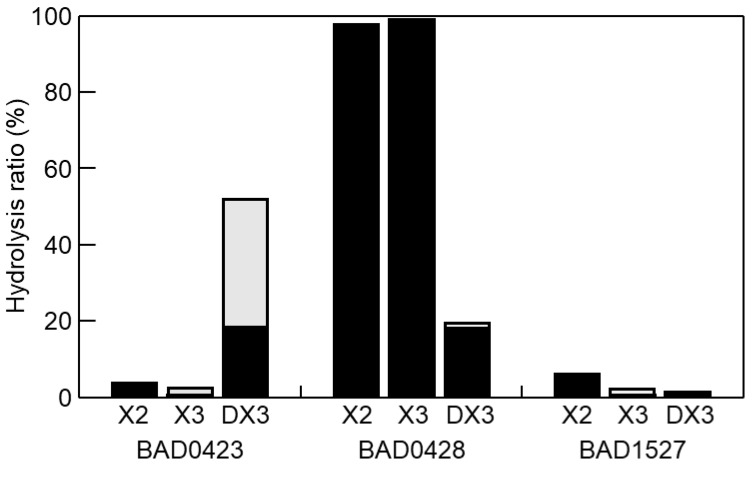
Hydrolysis products of oligosaccharides (XOS). Ten mM XOS was hydrolyzed by 50 μg/mL BAD0423, BAD0428, and BAD1527 at pH 6.5 and 37 °C for 1 h. The products were analyzed by HPLC. Black and gray indicate the amount of X1 and X2, respectively.

**Figure 6:**
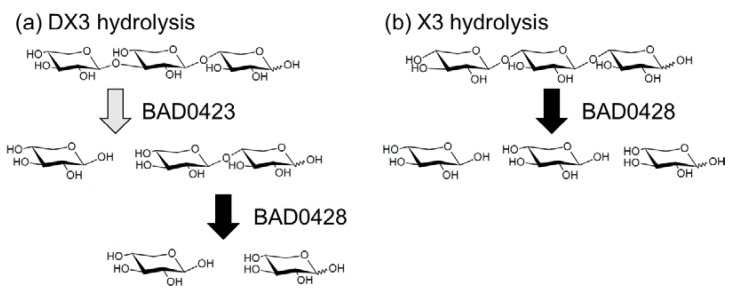
The putative hydrolysis mechanism of DX3 and X3. (**a**) DX3; (**b**) X3.

Although we selected four GH43 genes to evaluate the DX3 hydrolysis, the BAD1203 gene did not amplify by PCR. Therefore, we used three GH43 enzymes (BAD0423, BAD0428, and BAD1527). These enzymes were expressed as bacterial recombinant enzymes. The p-nitrophenyl-β-d-xylopyranoside (pNP-X) activity of BAD0423, BAD0428, and BAD1527 were 6, 77,035, and 10 mU/mg, respectively, showing that the characters of the enzymes were the same as previous reports [21,45,46,47]. Using these enzymes, hydrolysis products of X2, X3, and DX3 were evaluated (Figure 5). BAD0423 showed low activities on X2 and X3 but high activity on DX3. The hydrolysis ratio reached 52% for 1 h hydrolysis, and the hydrolysis products were X1 and X2. BAD0428 hydrolyzed XOS producing X1 as the main product. The activity on DX3 was weak (19%) as compared with X2 and X3. The low activity of BAD0428 on DX3 was due to the low hydrolysis activity on β-(1→3)-linkage at the nonreducing end since the hydrolysis products of DX3 was X1, meaning that the produced X2 was immediately hydrolyzed. BAD1527 weakly hydrolyzed XOS. The specific activity of BAD0423, BAD0428, and BAD1527 on DX3 was determined by HPLC as 2988, 1440, and 84 mU/mg, respectively. The hydrolysis products of BAD0423 on DX3 remained X2, indicating that the cooperative hydrolysis was necessary for complete DX3 hydrolysis. Therefore, BAD0423 and BAD0428 were used for DX3 hydrolysis, resulting in the hydrolysis ratio of 77%, which was slightly high hydrolysis by each enzyme (71%), and that of X1 was 90%. From these data, BAD0423 was the key enzyme for DX3, and the hydrolysis products contained X2, therefore, the cooperative hydrolysis with highly active β-xylosidase (BAD0428) were needed (Figure 6). 

## 3. Discussion

Red alga dulse possesses a unique xylan in the cell wall, and we have previously developed the preparation method of DX3 (β-(1→3)-xylosyl-xylobiose) [19]. In this study, we evaluated the prebiotic effect of DX3 on bacteria. Among the tested bacteria, *Bifidobacterium adolescentis* effectively metabolized DX3, indicating that the bacterium possessed the enzyme having β-(1→3)-xylosidase activity. Two candidate enzymes (BAD0423: GH43_12 and BAD0428: GH43_11) were obtained from bioinformatics analysis. We characterized these enzymes, revealing that BAD0423 was the key enzyme for the hydrolysis of β-(1→3)-xylosyl linkage, and BAD0428 was necessary for the complete hydrolysis of DX3 for X1. These genes were members of the xylooligosaccharide utilization cluster composed of xylose isomerase (BAD0422), GH43_12 arabinofuranosidase (BAD0423), LacI transcriptional regulators (BAD0424), solute binding protein (BAD0425), two ABC transporter (BAD0426 and BAD0427), GH43_11 β-xylosidase (BAD0428), two esterase (BAD0429 and BAD0430), and xylulose kinase (BAD0431) [48]. It has been reported that the solute binding protein from *Bifidobacterium animals* subsp. *lactis Bl-04* captures XOS and arabino-xylooligosaccharides with the specificity for tri- and tetrasaccharides, which show 69% identity with BAD0425 [22]. The structure of arabinoxylobiose was α-(1→3)-arabinofuranosyl xylobiose, which was quite similar to DX3, suggesting that DX3 would also be incorporated into *B*. *adolescentis* by the same solute binding protein (BAD0425). *Bifidobacterium* sp., which did not increase in XOS containing medium in this test, did not possess the cluster, indicating that the enzymes containing this cluster are necessary for DX3 hydrolysis.

The activities of BAD0423 (α-l-arabinofuranosidase) on pNP-Ara and pNP-X have been reported as 249 and 4 mU/mg, respectively [46], and we also obtained the same results. In addition, the DX3 activity of BAD0423 was 2988 mU/mg, which was 10 and 500 times higher than that of pNP-Ara and pNP-X, respectively. The arabinose releasing activity against arabinoxylan of AXH-m23 from *B*. *adolescentis* DSM 20083 (BAD0423) has been reported as 80,000 mU/mg [45], meaning that the favorite substrate at subsite-1 of BAD0423 would be α-(1→3)-arabinofuranose residues. The activity of the GH43_12 enzyme from *Streptomyces* sp. SWU10 showed β-(1→3)-xylosidase activity with the specific activity of 1330 mU/mg toward β-(1→3)-xylobiose, which was 440-fold higher than that of X2 [24]. The identity of this enzyme and BAD0423 was low (20%), suggesting that there are many enzymes having β-(1→3)-xylosidase activity in GH43_12. 

The growth rate of *B*. *adolescentis* in X3 and DX3 was almost the same, indicating that the utilization of X3 and DX3 corresponded to the enzyme activities. This means that the high amount of BAD0423 in the bacterium was needed as compared with BAD0428. A study on transcriptional analysis and proteome analysis in *Bifidobacterium animalis* sp. showed that the expression level of β-xylosidase (a homolog of BAD0428) was high [48,49,50], indicating that additional β-(1→3)-xylosidases were required. Although there are fewer reports on β-(1→3)-xylosidase activity, our results suggested that the enzyme having α-(1→3)-arabinofuranosidase activity also showed β-(1→3)-xylosidase activity. Therefore, we attempted to find the enzyme that corresponded with the *Bacteroides* sp. The common enzyme families having α-(1→3)-arabinofuranosidase were found in GH30, -43, and -51. 

In GH30, only subfamily 2 possessed β-xylosidase activity. Two enzymes from *B*. *vulgatus* showed 60% identity, but these showed a low identity to the *B*. *adolescentis* enzyme (27% and 30%). In addition, *B*. *thetaiotaomicron*, which showed the same growth rate with *B*. *vulgatus*, did not possess these subfamily enzymes. The GH30 enzyme from *Bifidobacterium breve* K-110 showed pNP-X activity but not pNP-Ara [51], indicating that high β-(1→3)-xylosidase activity was not expected in this family. 

The activities for DX3, on seven of the three GH43 enzymes, were characterized in this study. GH43_10 (BAD0301) has been reported as the double substituted xylan α-1,3-l-specific arabinofuranosidase [45,46]. GH43_27 from *B*. *longum* sp. (BLLJ_1852) has shown an arabinan-degrading exo-1,2-1,3-α-l-arabinofuranosidase [40] and the study showed that the activity of arabinose releasing from arabinoxylan of BLLJ_1852 was 0.62-fold of pNP-Ara, suggesting that the character did not agree with the high DX3 hydrolysis activity. In addition, they also mentioned that the subfamily 27 possessed various substrate specific enzymes. The identity of GH43_27 BAD0149 with BLLJ_1852 was 40%. However, two *Bacteroides* sp. did not possess this subfamily enzymes, suggesting a low possibility of high DX3 hydrolysis activity. GH43_26 BAD0152 was classified into exo-α-1,5-l-arabinofuranosidase, suggesting that this was not suitable. Although the main activity of GH43_11 BAD0428 was β-(1→4)-xylosidase activity, the enzyme also showed β-(1→3)-xylosidase activity. The activity of the GH43_11 enzyme from *Vibrio* sp. XY-214 has shown high β-(1→3)-xylosidase activity [23]. The identity of these enzymes was 34%. We attempted to express GH43_11 BAD1203 enzymes resulting in failure of characterization. The primary sequence identities of BAD1203 to BAD0423 and *Vibrio* sp. enzyme were 32% and 33%, respectively, remaining the potential for DX3 hydrolase. Although the activity of GH43_22 BAD1527 on the tested substrate was low, the high activity was reported on arabinogalactan from *B*. *longum* subsp. *longum* showing 20% identity [44]. Among GH43, the subfamily 11 (BAD1203) had potential for β-(1→3)-xylosidase activity, however only one *Bacteroides* sp. in the tested possessed this subfamily enzyme, indicating that alternative enzymes could exist. 

In GH51, BAD1205 and BAD1524, which was designated as α-l-arabinofuranosidase, showed low identity (13%). The two enzymes also showed low identities with four GH51 from *B*. *longum* subsp. *longum* JCM 1217 (11% to 18%). α-l-Arabinofuranosidase from *B*. *longum* B667, which possess 100% identity with BLLJ0445, showed high activity on the arabinose side chain of arabinan as compared with that of arabinoxylan [52]. The GH51 enzymes within *Bacteroides* sp. showed high identities as follows: BT0368 and BVU1001 (74%), BT3657 and BVU0496 (57%), and BT0438 and BVU4054 (77%). BAD1205 showed 27% and 29% identities with BT0348 and BVU4054, respectively, and BAD1524 showed 30% and 32% identities with BT3657 and BVU0496, respectively, suggesting the potential for DX3 hydrolysis. Studies on GH51 have shown the high activity on arabinan side chain than arabinoxylan [53,54,55], and pNP-Ara activity of GH51 was lower than that of GH43 [53]. Therefore, the activity of DX3 could be low. 

The activity of GH120 β-xylosidase has been reported as pNP-Ara and pNP-X [47]. When the enzyme hydrolyzed AXOS, only X1 was released but not Ara, suggesting that the possibility of DX3 hydrolysis activity of this enzyme was low. 

*Bacteroides vulgatus* also possesses XOS utilization locus, containing four GH43 enzymes [55]. The study reported that two GH43 enzymes (BVU_0039 and BVU_0040, subfamily 1) were sufficient for XOS utilization. *B*. *adolescentis* possesses the same subfamily 12 with BVU_0039, and the rest of subfamily BVU_0040 (subfamily 1) would work like BAD0428, a β-xylosidase. 

## 4. Materials and Methods 

### 4.1. Materials

Dulse (*Palmaria palmata* in Japan) was harvested near Usujiri, Hokkaido, Japan in February 2017 and stored at −30  °C until use [30,31]. Xylose, β-(1→4)-xylobiose (X2) and β-(1→4)-xylotriose (X3) were purchased from Wako Pure Chemical Industries (Osaka, Japan). β-(1→4)-Xylotetraose (X4) and β-(1→4)-xylopentaose (X5) were purchased from Megazyme (Bray, Ireland). All other reagents were purchased from Wako Pure Chemical Industries (Osaka, Japan). 

### 4.2. Preparation of DX3 from Red Alga Dulse

DX3 (2^3^-β-d-xylosyl-xylobiose) (99.0%>) was prepared as previously in [19]. The frozen dulse thalli were lyophilized and homogenized by a Wonder Blender WB-1 (OSAKA CHEMIKAL CO., Osaka, Japan) into powder. The dulse powder was suspended in 40 volumes (v/w) of distilled water, and the suspension was autoclaved at 121  °C for 20  min. Then, the solution was centrifuged at 15,000× g for 10  min. The supernatant was dialyzed against distilled water with a dialysis tube (molecular weight cut off, approximately 14 kDa, EIDIA Co., Ltd., Tokyo, Japan). The dialysis solution was centrifuged at 15,000× g for 10 min to remove small amount of insoluble materials. The supernatant (10  mg/ml) was hydrolyzed for 6  h by 2 wt% (5 U) of SucraseX (Mitsubishi-Chemical Foods Corporation, Japan) at pH 6.0 and 50  °C. The enzyme reactions were stopped by heating at 100  °C for 10  min. The products were applied to an activated carbon column (φ 3 × 35 cm) pre-equivalated with distilled water. The carbohydrate was eluted by a stepwise system consisting of 0%, 10%, 15%, 20%, and 25% ethanol. The DX3 fractions were detected by HPLC equipped with a Sugar-D column and refractive index detector (RI). The DX3 fractions were pooled, evaporated, and lyophilized as DX3 powder. The purity of DX3 was evaluated by HPLC. 

### 4.3. Bacterial Growth 

The bacterial strains used in this study (Bacteroides thetaiotaomicron JCM 5827, Bacteroides vulgatus JCM 5826, Bifidobacterium adolescentis JCM 7046, Bifidobacterium longum subsp. infantis JCM 1222, Bifidobacterium longum subsp. longum JCM 1217, Clostridium paraputrificum JCM 1293, Eubacterium limosum JCM 6421, Lactobacillus acidophilus JCM 1132, and Lactobacillus casei JCM 1134) were provided by Japan Collection of Microorganisms, RIKEN BRC which is participating in the National BioResource Project of the MEXT, Japan. The defibrinated blood was purchased from Cosmo Bio CO., LTD. 

PYF medium consisted of 10 g Trypticase Peptone (BBL, Becton Dickinson & Company, Cockeysville, MD, USA), 5 g yeast extract (Difco Lab, Detroit, MI, USA), 0.5 g L-cysteine hydrochloride, 40 mL Fildes solution, and 40 mL salt solution per 1 L. Fildes solution contained 0.85% NaCl, 6 mL HCl, 50 mL horse defibrinated blood, and pepsin 1:10,000 (Fujifilm Wako Pure Chemical Industries, Ltd., Osaka, Japan). The salt solution contained 0.2 g CaCl_2_, 0.2 g MgSO_4_ (7H_2_O), 1.0 g KH_2_PO_4_, 1.0 g K_2_HPO_4_, 10 g NaHCO_3_, and 2.0 g NaCl in 1 L deionized water. Before the fermentation trials, bacterial strains were grown on GAM plate (Nissui Pharmaceutical, Japan) and then precultured in PYF broth containing 1% glucose.

Precultured bacterial suspensions were prepared at a concentration of 1 × 10^7^ CFU/tube in the PFY medium containing 0.5% carbohydrates (glucose, xylose, X3, and DX3), and then incubated at 37 °C for 96 h in anaerobiosis condition (Anaeropack Anaero, Mitsubishi Gas Chemical Co., Inc., Germany). The growth of bacteria in each carbohydrate was evaluated as ΔpH and ΔOD_600_ by the lowering of pH and the increase in OD_600_ by subtraction of the values without carbohydrate. 

Time course growth of *B*. *adolescentis* and *B*. *vulgatus* was monitored up to 32 h and the OD_600_ was measured at 24, 28, and 32 h. All the growth assays were performed in triplicate.

### 4.4. Bioinformatics 

The genes related to carbohydrate-active enzymes in bacteria were collected from the CAZy database (http://www.cazy.org/). Among them, the glycoside hydrolase families containing β-xylosidase genes (EC 3.2.1.37) were selected. The function of genes that classified into GH containing several enzymes was manually confirmed by Blast search (https://blast.ncbi.nlm.nih.gov/Blast.cgi). Sequences in the same family were confirmed by CLUSTALW (https://www.genome.jp/tools-bin/clustalw). The bacterial genomic sequences used in this study were as follows: *B*. *thetaiotaomicron* JCM 5827 (GenBank AE015928), *B*. *vulgatus* JCM 5826 (GenBank CP000139), *B*. *adolescentis* JCM 7046 (GenBank LNKM00000000), *B*. *longum* subsp. *infantis* JCM 1222 (GenBank AP010889), *B*. *longum* subsp. *longum* JCM 1217 (GenBank AP010888), *C. paraputrificum* JCM 1293 (GenBank UAWH00000000), *E*. *limosum* JCM 6421 (GenBank CP019962), *L*. *acidophilus* JCM 1132 (GenBank AZCS00000000), and *L*. *casei* JCM 1134 (GenBank AP012544). 

### 4.5. Cloning, Expression, and Purification of Enzymes

Genomic DNA from *B*. *adolescentis* JCM 7046 was extracted using an innu PREP Bacteria DNA Kit (Analytik Jena, Jena, Germany). We prepared the full length of xylosidase constructs (BAD0423, BAD0428, and BAD1527) having the His-tag at N-terminus, since these did not possess signal peptide (http://www.cbs.dtu.dk/services/SignalP-4.1/). The xylosidase genes were amplified from the genomic DNA by PCR using KOD FX new DNA polymerase (Toyobo, Japan) with the sets of primers (Table S2). The PCR products were cloned into the pBluescript II SK(+) vector and sequenced. The fragments were digested by restriction enzymes (Table S1) and ligated into pET28a vector with the digestion of the sets of restriction enzymes. *Escherichia coli* BL21 (DE3) was transformed with the expression plasmid. Protein expression and purification were performed as previously [56]. The protein expression was induced by 0.1 mM isopropyl β-d-thiogalactopyranoside. The cells were collected by centrifugation and disrupted by sonication. The recombinants were purified by TALON metal affinity resin (Talon, Clontech, Japan) according to the manufacturer’s protocol. The active fractions were dialyzed with a dialysis tube (molecular weight cutoff about 14 kDa, EIDIA Co., Ltd., Tokyo, Japan) against 20 mM sodium phosphate buffer (pH 6.5). The purity of recombinants was confirmed as a single band by SDS-PAGE (Figure S1). The protein concentrations were determined by the absorption at 280 nm using molecular absorptivity of each protein.

### 4.6. Activity Assay

β-d-Xylosidase activity was measured based on the release of *p*-nitrophenyl (pNP) from pNP-β-d-Xylopyranoside (pNP-X). The reaction mixture containing 18.4 mM pNP-X, an appropriate amount of purified enzyme, and 10 mM sodium phosphate buffer (pH 6.5) was incubated at 37 °C for 30 min. The reaction was terminated by adding 1.5 volumes of 1.0 M Na_2_CO_3_, and the released pNP was measured at 400 nm. One unit was defined as the amount of enzyme that released 1.0 μmol pNP per minute.

### 4.7. HPLC Analysis

Hydrolysis products of XOS were determined by HPLC using a RI detector. The enzyme reaction was performed as follows: 10 mM XOS (X2, X3, and DX3) were hydrolyzed by 50 μg/mL or enzymes in 20 mM sodium phosphate buffer (pH 6.5) at 37 °C for 1 h. The reaction was terminated by heating at 95 °C for 5 min. The products were applied to HPLC equipped with a Sugar-D column (4.6  ×  250  mm, Nakalai Tesque, Kyoto, Japan) with the column oven temperature at 40 °C. The products were eluted with an isocratic elution system of acetonitrile/water (4:1, v/v) at a flow rate of 1.0  ml/min. The enzyme activities were determined by the amount of hydrolysis products. Xylose and XOS were used as standards.

## 5. Conclusions

*Bifidobacterium adolescentis* utilized DX3 from red alga dulse similar to X3. Therefore, key enzymes for β-(1→3)-xylosidase activity were investigated, with the result that BAD0423 (GH43_12) and BAD0428 (GH43_11) were required for DX3 hydrolysis. Although xylose was released from DX3 by the above two enzymes, the rate of xylose release from X3 was faster than DX3, which did not coincide with the growth rate of *B*. *adolescentis*. In search of enzymes, GH43_11 and GH51 enzymes occurred as candidates. However, the putative activity for DX3 could be low. Therefore, cooperative hydrolysis of a repertoire of various α-l-arabinofuranosidases are needed. 

## Supplementary Materials

The following are available online at https://www.mdpi.com/1660-3397/18/3/174/s1, Table S1: Effect of carbohydrate on bacterial growth and pH, Table S2: Primers used in this study, Figure S1: SDS-PAGE of recombinant proteins.
