# Supplementary material for: Identification of a Key Enzyme for the Hydrolysis of β-(1→3)-Xylosyl Linkage in Red Alga Dulse Xylooligosaccharide from Bifidobacterium Adolescentis
Source: Mar Drugs. 2020 Mar 20;18(3):174. doi: 10.3390/md18030174 (PMC7142710; doi:10.3390/md18030174)
Supplement: Supplementary file 1 [file marinedrugs-18-00174-s001.zip › 726312 SI to conversion/marinedrugs-726312 table S1.pdf]

**Table S1**. Effect of saccharide on bacterial growth and pH

| Bacteria | JCM | | G1* | X1* | X3* | DX3* |
|---|---|---|---|---|---|---|
| *Bacteroides thetaiotaomicron* | 5827 | $OD_{600}$ | 0.93±0.01 | 1.01±0.02 | 0.33±0.02 | 0.24±0.01 |
| | | pH | -0.80±0.15 | -1.07±0.21 | -0.13±0.08 | -0.26±0.05 |
| *Bacteroides vulgatus* | 5826 | $OD_{600}$ | 0.17±0.01 | 0.40±0.01 | 0.43±0.03 | 0.36±0.03 |
| | | pH | -1.13±0.04 | -1.35±0.12 | -1.25±0.03 | -1.04±0.02 |
| *Bifidobacterium adolescentis* | 7046 | $OD_{600}$ | 1.46±0.00 | 1.10±0.00 | 1.34±0.02 | 1.39±0.01 |
| | | pH | -2.34±0.10 | -2.29±0.08 | -2.23±0.06 | -2.37±0.08 |
| *Bifidobacterium longum* subsp. *infantis* | 1222 | $OD_{600}$ | 1.28±0.04 | 0.00±0.00 | -0.01±0.01 | 0.02±0.02 |
| | | pH | -2.15±0.04 | -0.02±0.05 | 0.06±0.04 | 0.03±0.07 |
| *Bifidobacterium longum* subsp. longum | 1217 | $OD_{600}$ | 1.59±0.01 | 1.01±0.01 | -0.01±0.00 | 0.00±0.00 |
| | | pH | -2.39±0.05 | -2.37±0.17 | 0.17±0.07 | 0.17±0.15 |
| *Clostridium paraputrificum* | 1293 | $OD_{600}$ | 1.55±0.03 | 0.02±0.03 | N.D.** | 0.03±0.03 |
| | | pH | -1.49±0.07 | 0.07±0.07 | N.D.** | -0.01±0.04 |
| *Eubacterium limosum* | 6421 | $OD_{600}$ | 1.40±0.00 | -0.03±0.01 | N.D.** | 0.02±0.00 |
| | | pH | -1.43±0.01 | -0.04±0.21 | N.D.** | 0.04±0.10 |
| *Lactobacillus acidophilus* | 1132 | $OD_{600}$ | 0.88±0.03 | -0.00±0.00 | -0.01±0.01 | 0.01±0.00 |
| | | pH | -2.34±0.06 | -0.08±0.14 | 0.01±0.07 | 0.03±0.11 |
| *Lactobacillus casei* | 1134 | $OD_{600}$ | 0.77±0.01 | 0.01±0.01 | 0.00±0.00 | 0.02±0.01 |
| | | pH | -2.59±0.07 | 0.02±0.12 | 0.06±0.15 | 0.09±0.06 |
| *Lactobacillus salivarius* | 1040 | $OD_{600}$ | 1.19±0.02 | -0.02±0.01 | 0.03±0.02 | 0.05±0.02 |
| | | pH | -2.55±0.06 | -0.08±0.02 | 0.18±0.02 | 0.10±0.06 |

The $OD_{600}$ and pH were monitored after incubation in PYF medium with or without sugar at 37 ℃ for 96 h.

* The data were obtained by the subtraction of each bacterial growth without sugar.

** N.D., Not determined.
