# Supplementary material for: Identification of a Key Enzyme for the Hydrolysis of β-(1→3)-Xylosyl Linkage in Red Alga Dulse Xylooligosaccharide from Bifidobacterium Adolescentis
Source: Mar Drugs. 2020 Mar 20;18(3):174. doi: 10.3390/md18030174 (PMC7142710; doi:10.3390/md18030174)
Supplement: Supplementary file 1 [file marinedrugs-18-00174-s001.zip › 726312 SI to conversion/Marinedrugs-726312 table S2.pdf]

Table S2. Primers used in this study

Primer	Sequence	Target genes	GH
BAD0423GH43_12	5'-CATATGACCACAACGATTACCATCGCC-3'	BAD0423	GH43_12
BAD0423GH43_12	5'-GGATCCCTATGCCATGAAGCCGGCCATG-3'		
BaGH43Fw	5'-CATATGAAGATTTCCAACCCGGTGCTCACC-3'	BAD0428	GH43_11
BaGH43Re	5'-AAGCTTCTACCGGTTGTCGGGAAGCTCCC-3'		
GH43_BAD1527F	5'-CATATGAGCATCGACACCCAGGCCTATC-3'	BAD1527	GH43_22
GH43_BAD1527R	5'-AAGCTTCTACAGGGCGAAGGCGGTTTCG-3'		
GH43_BAD1203F	5'-CATATGCTGCATAATCCGATCTTCAAAGGC-3'	BAD1203	GH43_11
GH43_BAD1203R	5'-AAGCTTTCAGTCGACGGGCTTCGTCTCG-3'		

Underline shows restriction enzyme site.
