# Supplementary figures and images for: Identification of a Key Enzyme for the Hydrolysis of β-(1→3)-Xylosyl Linkage in Red Alga Dulse Xylooligosaccharide from Bifidobacterium Adolescentis
Source: Mar Drugs. 2020 Mar 20;18(3):174. doi: 10.3390/md18030174 (PMC7142710; doi:10.3390/md18030174)

Supplementary materials: Figure S1

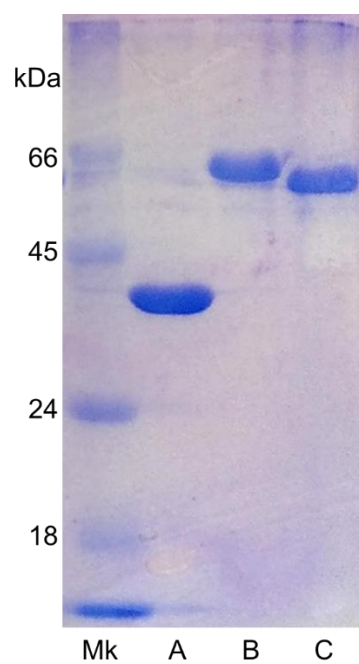

**Figure S1.** SDS-PAGE. (A), BAD1527; (B), BAD0423; and (C) BAD0428.

Supplement: Supplementary file 1 [file marinedrugs-18-00174-s001.zip › 726312 SI to conversion/marinedrugs-726312 SI figures.pdf]
